# Genetic association analysis of dietary intake and idiopathic pulmonary fibrosis: a two-sample mendelian randomization study

**DOI:** 10.1186/s12890-023-02831-8

**Published:** 2024-01-05

**Authors:** Chenwei Zhang, Yujia Xi, Yukai Zhang, Peiyun He, Xuesen Su, Fangfang Fan, Min Wu, Xiaomei Kong, Yiwei Shi

**Affiliations:** 1 Department of Respiratory and Critical Care Medicine, Shanxi Medical University Affiliated First Hospital, Taiyuan, 030000 China; 2 NHC Key Laboratory of Pneumoconiosis, Taiyuan, 030000 China; 3 Department of Urology, The Second Hospital of Shanxi Medical University, Taiyuan, 030000 China; 4 First School of Clinical Medicine, Shanxi Medical University, Taiyuan, 030000 China

**Keywords:** Idiopathic pulmonary fibrosis, Dietary intake, Mendelian randomization, Causal relationship, Prevention strategy

## Abstract

**Background:**

IPF is a complex lung disease whose aetiology is not fully understood, but diet may have an impact on its development and progression. Therefore, we investigated the potential causal connection between dietary intake and IPF through TSMR to offer insights for early disease prevention recommendations.

**Methods:**

The study incorporated 29 dietary exposure factors, oily fish intake, bacon intake, processed meat intake, poultry intake, beef intake, pork intake, lamb/mutton intake, non-oily fish intake, fresh fruit intake, cooked vegetable intake, baked bean intake, fresh tomato intake, tinned tomato intake, salad/raw vegetable intake, Fresh fruit intake, coffee intake, tea intake, water intake, red wine intake, average weekly beer plus cider intake, alcoholic drinks per week, cereal intake, bread intake, whole-wheat intake, whole-wheat cereal intake, cheese intake, yogurt intake, salt added to food and whole egg intake. The study explored the causal link between diet and IPF using TSMR analysis, predominantly the IVW method, and performed sensitivity analyses to validate the results.

**Result:**

The study revealed that consuming oily fish, yogurt, and dried fruits had a protective effect against IPF, whereas the consumption of alcoholic beverages and beef was linked to an increased risk of IPF.

**Conclusion:**

In this MR study, it was discovered that the consumption of oily fish, yogurt, and dried fruits exhibited a protective effect against IPF, whereas the intake of alcoholic beverages and beef was associated with an elevated risk of IPF. These findings underscore the significance of making informed and timely dietary decisions in IPF prevention.

**Supplementary Information:**

The online version contains supplementary material available at 10.1186/s12890-023-02831-8.

## Introduction

Idiopathic pulmonary fibrosis (IPF) is a chronic and progressive respiratory disease. It is distinguished by the formation of fibrous tissue in the lungs, which leads to irreversible lung dysfunction [1]. IPF has varying occurrence and prevalence rates, ranging from 0.09 to 1.30 and 0.33 to 4.51 per 10,000 individuals, respectively. It predominantly affects males over 50 years old and exhibits significant geodiversity [2]. Given the intricate nature of the disease, it is imperative to distinguish between interstitial lung diseases (ILDs) that share clinical features with IPF, and the inherent uncertainty in specific imaging features indicating IPF. The result is a stark prognosis of a mere 3–5 years of survival for diagnosed IPF patients [3, 4]. Currently, the primary treatments for IPF are predominantly centered around oxygen therapy, pulmonary rehabilitation, and palliative care. While pirfenidone can help slow disease progression as a drug therapy for IPF, it also suffers from high cost and potential side effects [5]. In conclusion, the lack of clarity regarding the causative factors of IPF, coupled with its substantial disease burden and limited treatment options, has sparked an increasing interest in investigating how specific dietary patterns and individual components may contribute to disease prevention [6].

Numerous studies highlight the significance of dietary prevention in maintaining good health, as informed dietary choices aid in controlling the risk of IPF [6–8]. Evidence suggests that consuming antioxidant-rich foods, such as blueberries, citrus fruits, and others, can alleviate oxidative stress and inflammation, potentially preventing and reducing the risk of IPF [9, 10]. Consuming foods high in polyunsaturated fatty acids, particularly Omega-3 fatty acids, is believed to partially slow the progression of idiopathic pulmonary fibrosis (IPF) by reducing inflammation and abnormal immune reactions [11]. Furthermore, there is evidence of an inverse relationship between vitamin D deficiency and reduced lung function in IPF patients, indicating that dairy products are a valuable dietary choice for prevention [12, 13]. Nutritional epidemiological studies have demonstrated correlations between diet and IPF, however, the challenges in controlling variables and retrospective design emphasize the pressing need for rigorous scientific methodologies to establish accurate causal relationships [14].

Mendelian randomization is an approach that examines the causal relationship between exposures and outcomes by using genetically correlated variants that directly affect outcomes as instrumental variables for exposures [15]. MR offers the advantage of bypassing reverse causation and reducing confounding factors compared to retrospective studies [16]. It also has broader applicability, increased resistance to ethical concerns, shorter study timelines, and decreased costs compared to randomized controlled trial (RCT) [17]. Given the rarity of IPF and the limited patient population, there is a crucial need to explore disease-influencing factors through MR analysis of publicly available GWAS data. A prior TSMR study indicates that a heightened circulating IL-14 level is a significant risk factor for IPF [18]. This study utilizes a two-sample Mendelian randomization approach to examine the causal relationship between 29 specific dietary factors and IPF. The study’s findings address gaps in current causal knowledge and provide corroborative evidence for scientifically-based dietary strategies in the prevention and management of IPF.

## Method and result

### Study design

To investigate the causal relationship between different dietary intake types and the development and intensity of IPF, this TSMR study included 29 specific dietary categories, such as liquid consumption, meat intake, fruit and vegetable consumption, and cereal intake, that were treated as exposures (Table 1). To ensure the strength of our findings, the selected genetic instrumental variables must adhere to three fundamental assumptions: firstly, they should display a strong correlation with the identified dietary factors; secondly, they must remain unassociated with established and potential confounding variables; and thirdly, their effects should be limited exclusively to the relationship between the particular dietary exposures and the outcomes of IPF, without being affected by other likely confounding factors. This study employs STROBE-MR (Strengthening the Reporting of Observational Studies in Epidemiology using Mendelian Randomization) Table to enhance the clarity of our research process presentation.

**Table 1:** Sources of information on GWAS data related to dietary intake and IPF in MR analysis

GWAS ID	Exposure and outcome	Number of SNPs	F-statistic mean	Sample size
ukb-b-10,565	Tinned tomato intake	21	11.99	64,949
ukb-b-11,348	Bread intake	21	38.54	452,236
ukb-b-14,179	Lamb/mutton intake	24	17.76	460,006
ukb-b-1489	Cheese intake	43	40.13	451,486
ukb-b-14,898	Water intake	27	26.17	427,588
ukb-b-15,926	Cereal intake	26	29.33	441,640
ukb-b-16,576	Dried fruit intake	19	22.30	421,764
ukb-b-17,627	Non-oily fish intake	8	28.15	460,880
ukb-b-1996	Salad / raw vegetable intake	13	14.61	435,435
ukb-b-2209	Oily fish intake	41	38.93	460,443
ukb-b-2299	Green bean intake	27	16.45	64,949
ukb-b-2375	Whole-wheat cereal intake	6	11.26	64,949
ukb-b-2862	Beef intake	8	26.11	461,053
ukb-b-3881	Fresh fruit intake	33	15.52	446,462
ukb-b-4058	Baked bean intake	24	12.48	64,949
ukb-b-4075	Whole egg intake	11	14.26	64,949
ukb-b-5174	Average weekly beer plus cider intake	13	19.94	327,634
ukb-b-5237	Coffee intake	26	24.94	428,860
ukb-b-5640	Pork intake	10	17.96	460,162
ukb-b-6066	Tea intake	30	41.87	447,485
ukb-b-6324	Processed meat intake	16	38.68	461,981
ukb-b-730	Fresh tomato intake	18	46.90	64,949
ukb-b-7753	Yogurt intake	12	21.00	64,949
ukb-b-8006	Poultry intake	4	24.08	461,900
ukb-b-8089	Cooked vegetable intake	11	20.37	448,651
ukb-b-8121	Salt added to food	70	37.53	462,630
ukb-b-13,556	Red wine intake	1	50.28	64,949
ukb-b-4414	Bacon intake	3	19.42	64,949
ieu-b-73	Alcoholic drinks per week	26	70.99	335,394
ebi-a-GCST90018120	Idiopathic pulmonary fibrosis	NA	NA	1369

### Data sources

The GWAS summary-level data for this study were obtained from the IEU Open GWAS project (https://gwas.mrcieu.ac.uk), which consists of 18 batches primarily focused on the UK Biobank and FinnGen dataset, which are the two largest GWAS data biobanks in the world today [19]. The study included different dietary factors such as consumption of meat (intake of oily fish, bacon, processed meat, poultry, beef, pork, lamb/mutton, and non-oily fish) and fruits and vegetables (fresh fruit, cooked vegetables, baked beans, fresh tomato, and canned tomato). salad and raw vegetable consumption, fresh fruit consumption, beverages (coffee, tea, water, red wine, average weekly beer, and cider consumption, alcoholic drinks per week), basic foods (cereal, bread, Whole-wheat cereal), dairy products (cheese, yogurt), and other (added salt, whole egg consumption). Dietary data were obtained mainly from the Benjamin Neale Laboratory (http://www.nealelab.is/uk-biobank/) and the study conducted by Cole et al. Using Food Frequency Questionnaires (FFQ), this study analyzed individual food items as carefully curated quantitative traits (FI-QTs) and derived diet patterns from Principal Components (PC-DPs) using linear mixed models to conduct heritability and GWAS analyses. Technical term abbreviations such as FI-QTs and PC-DPs are explained at first use. Association tests were conducted utilizing linear regression models for each dietary habit while adjusting for age, age squared, sex, interactions between age and sex, and interactions between age squared and sex, in addition to the top 20 principal components of genotypes [20]. The GWAS dataset for IPF, identified by a primary or secondary ICD10 code from Hospital Episodes Statistics - specifically diagnosis code J84.1 - was obtained from the UK Biobank. The dataset comprised 1369 patients (with 1133 being unrelated participants) and 435,866 healthy controls. The data included 16,137,102 SNPs and was particularly focused on the European population [21].

### IV selection

To fulfill the three underlying assumptions of the chosen instrumental variable (IV), P-values less than 5.0 × 10^−8^ and 1.0 × 10^−5^ were initially utilized in this study. The objective was to identify the correct number of SNPs while reducing the possibility of false-positive associations. A smaller r^2^ value (r^2^ < 0.001) was selected to guarantee a very low genetic correlation among the selected instrumental variables (IVs). Choosing a window size of 10,000 kb (excluding palindromic structures) aims to maintain a uniform distribution of independent variables while reducing potential collinearity [22]. Additionally, establishing a threshold for F-values above 10 reduces bias from weak independent variables and ensures a robust association between IVs and exposure factors. With Phenoscanner, it is possible to verify a correlation between instrumental variables (IVs) and dietary factors without directly correlating with idiopathic pulmonary fibrosis (IPF) [22].

### Statistical analysis

The study primarily employs the inverse variance weighting (IVW) method, which combines multiple instrumental variables to calculate causal effects comprehensively, thus minimizing potential bias in individual IVs. Additionally, the study uses the Wald ratio method for single SNP analysis. The Weighted Median and MR-Egger methods augment the IVW methods and can address bias, correlation, and outliers among instrumental variables (IVs). Both the IVW and MR-Egger models utilized Cochran’s Q-test (*p* < 0.05) to evaluate heterogeneity. Leave-One-Out (LOO) plots were used to pinpoint influential outliers by progressively removing individual SNPs and re-estimating the causal effects. If the intercept of the MR-Egger regression significantly deviates from zero (*p* < 0.05), it implies the presence of pleiotropy. The MR-PRESSO approach identifies plausible pleiotropy and outlying observations, mitigating the likelihood of bias by carrying out outlier analysis and regression after removing outlying observations.

All analyses were conducted using R Version 4.3.1, and we employed the R packages “TwosampleMR” and “MRPRESSO” for these analyses.

## Result

### Characteristics of the IVs

The study assessed the causal link between 29 dietary factors and IPF by conducting an examination. A total of 744 SNPs were employed after excluding potential confounding factors such as smoking and outliers. The range of SNP utilization varied from 1 to 87. The average F-statistic value of 29.86 ensured a robust association between the selected IVs and the exposure under investigation.

### Causal relationship between dietary intake and risk of developing IPF

**Fig. 1:**
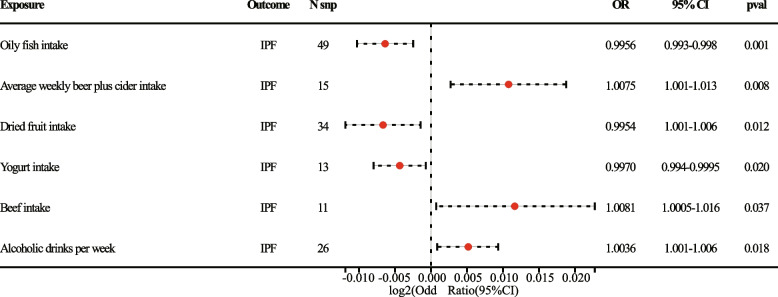
The forest plot showed primary results of the causal associations between dietary intake and IPF

**Fig. 2:**
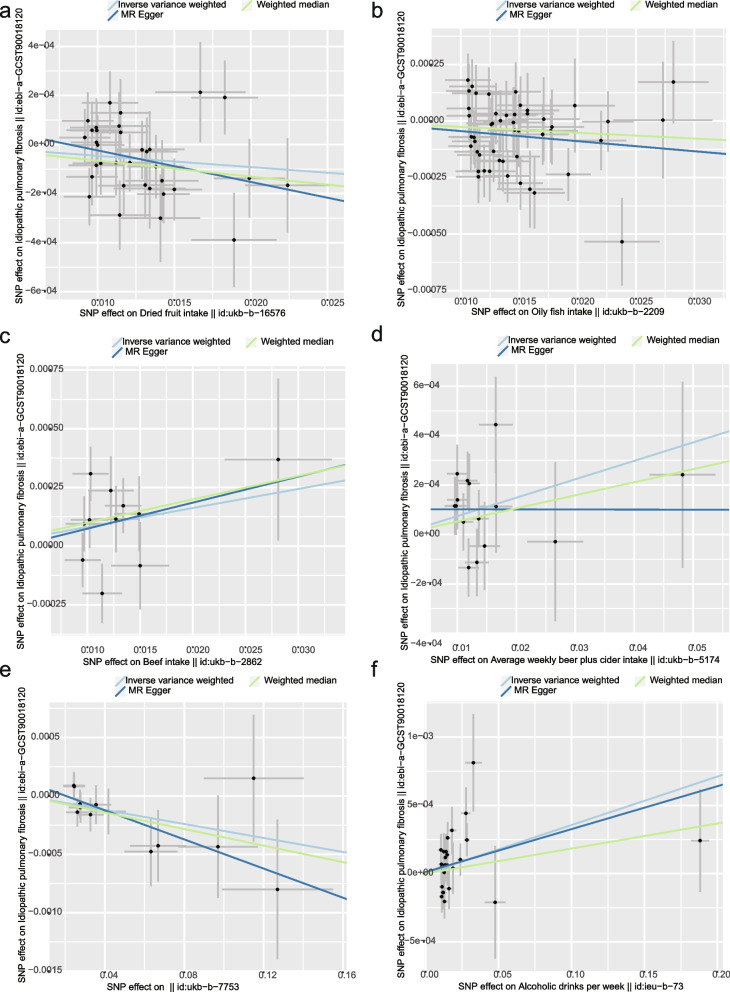
Scatter plots for the causal association between dietary intake and IPF (**a** Dried fruit intake, **b** Oily fish intake, **c** Beef intake, **d** Average weekly beer plus cider intake, **e** Yogurt intake, **f** Alcoholic drinks per week)

A total of six causal outcomes were revealed in this study (*P* < 0.05 for the IVW method). Oily fish intake (OR = 0.995, 95%CI = 0.993–0.998, *P* = 0.001), Yogurt intake (OR = 0.997, 95%CI = 0.994–0.9995, *P* = 0.020), and Dried fruit intake (OR = 1.004, 95%CI = 1.001–1.006, *P* = 0.018) could reduce the risk of developing IPF. The results were further validated using the Weighted Median method for Yogurt intake (OR = 0.996, 95% CI = 0.993–0.9998, *P* = 0.040) and Dried fruit intake (OR = 1.012, 95% CI = 0.988–0.999, *P* = 0.015). Average weekly beer plus cider intake (OR = 1.007, 95%CI = 1.001–1.013, *P* = 0.008), Beef intake (OR = 1.008, 95%CI = 1.0005–1.016, *P* = 0.037) and Alcoholic drinks per week (OR = 1.004, 95%CI = 1.001–1.006, *P* = 0.018) were risk factors for IPF. The Weighted Median results for Beef intake (OR = 1.010, 95% CI = 1.0005-1.020, *P* = 0.040) were also consistent (Figs. 1 and 2). Results from additional dietary MR analyses are provided in the supplementary materials (Supplementary Table 1).

### Sensitivity analysis of positive results

**Fig. 3:**
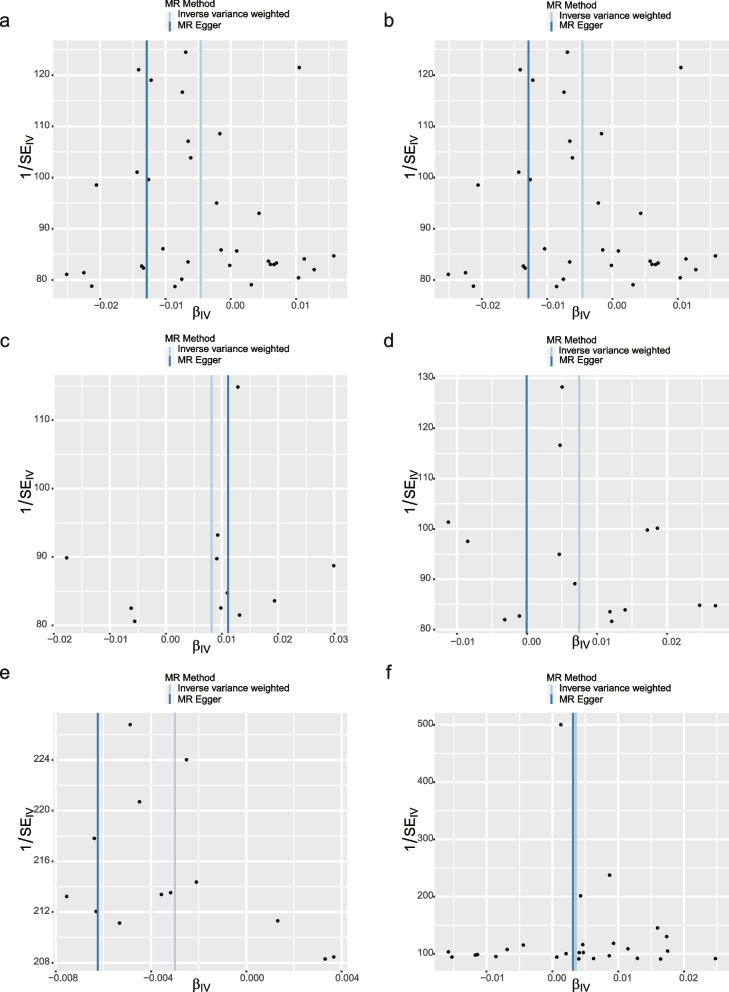
Funnel plots for the causal association between dietary intake and IPF (**a** Dried fruit intake, **b** Oily fish intake, **c** Beef intake, **d** Average weekly beer plus cider intake, **e** Yogurt intake, **f** Alcoholic drinks per week)

**Fig. 4:**
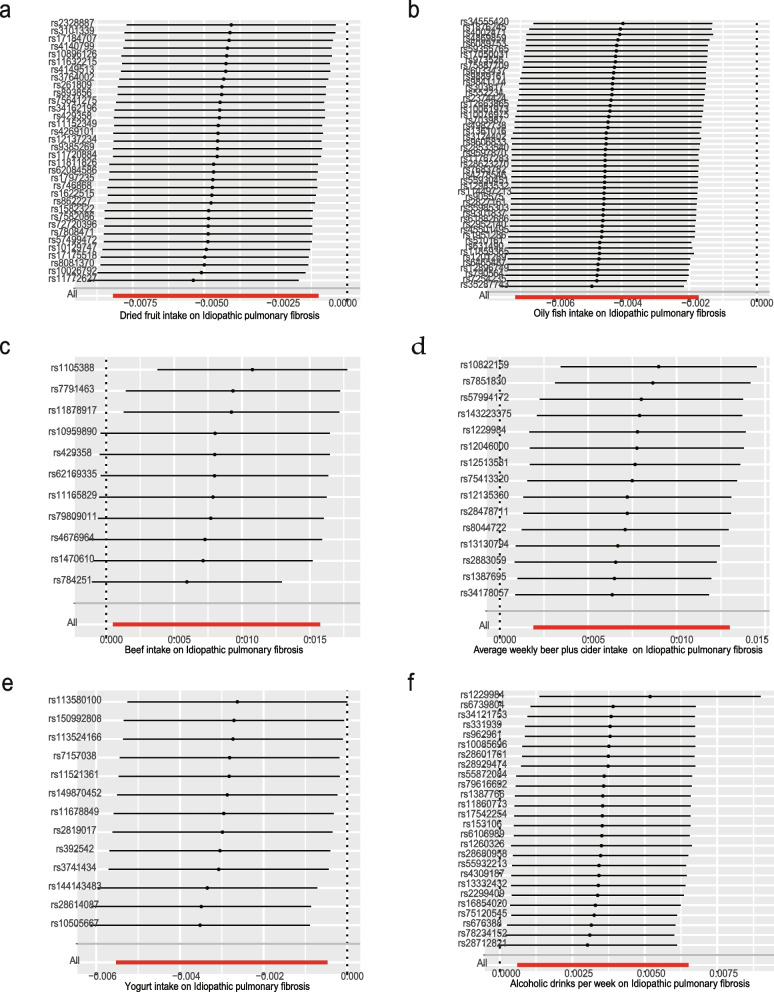
Leave-one-out plots for the causal association between dietary intake and IPF (**a** Dried fruit intake, **b** Oily fish intake, **c** Beef intake, **d** Average weekly beer plus cider intake, **e** Yogurt intake, **f** Alcoholic drinks per week)

For favorable outcomes, we excluded rs76640332 in the analysis of weekly alcohol consumption using MR-PRESSO and MR-Egger intercept analyses to address possible multiple effects. Furthermore, the lack of outcome heterogeneity was suggested by Cochrane’s Q p-values exceeding 0.05 (Table 2). The instrumental variables displayed symmetrical attributes in the funnel plots (Fig. 3). No outliers were identified in the study based on the analysis using the Leave-One-Out plot, which enhances the robustness and credibility of the findings (Fig. 4).

**Table 2:** MR estimates and sensitivity analyses of the causal relationship between dietary intake and IPF

**Exposure**	**Heterogeneity**		**MR-Egger regression**			**MR-PRESSO**
	MR Egger	IVW	Intercept	SE	*P*	*P*
**Dried fruit intake**	0.562	0.557	0.0001	0.0001	0.306	0.532
**Oily fish intake**	0.212	0.243	3.08E-7	8.32E-5	0.997	0.155
**Beef intake**	0.157	0.274	-3.68E-5	0.0002	0.873	0.833
**Average weekly beer plus cider intake**	0.356	0.367	0.0001	0.0001	0.381	0.238
**Yogurt intake**	0.896	0.853	0.0001	0.0001	0.259	0.456
**Alcoholic drinks per week**	0.086	0.108	1.12E-5	4.92E-5	0.822	0.131

## Discussion

This TSMR study is the first attempt to examine systematically the causal link between varied dietary intakes and the onset and severity of IPF. The overall objective is to provide evidence-based recommendations on dietary intake for early prevention among populations at risk of developing IPF. After removing smoking-related SNPs for the second phenotype using PhenoScanner and excluding anomalous outliers via the MR-PRESSO method, the study found no evidence of heterogeneity or pleiotropy. Oily fish intake, yogurt intake, and dried fruit intake demonstrated protective effects by lowering the risk of IPF. In contrast, weekly beer and cider intake, beef intake, and alcoholic beverage intake showed positive associations with the incidence of the disease.

Subsequently, we conducted a comparative analysis of studies addressing the same subject to validate the credibility of our findings. In terms of the impact of oily fish consumption on IPF, our results align with a comparable prior observational study, which found that the intake of oily fish played a role in protecting rat lung tissue against inflammation and fibrosis induced by MCT [23]. In a study on nutritional prevention of IPF, a significant association was found between the second and third quartiles of fruit consumption and a reduced risk of developing IPF. In contrast, our study established a causal relationship between dried fruit intake and IPF [8]. Multiple studies have indicated that alcohol intake can contribute to hepatic fibrosis through apoptosis, hepatic stellate cell activation, and inflammatory responses [24–26]. While the link between alcohol and pulmonary fibrosis has been less extensively studied, our study has identified an elevated incidence of IPF associated with alcohol intake, highlighting a potential focus for future IPF prevention efforts. No direct association with IPF was identified for yogurt and beef intake; therefore, we expanded our analysis to encompass additional studies and potential mechanisms to complement our findings.

We identified a negative correlation between the consumption of yogurt and an elevated risk of IPF. However, there was no evidence to suggest that cheese, another dairy product, contributes to IPF development. Yogurt contains higher amounts of probiotics such as active probiotics and lactobacilli compared to cheese. These bacteria are known to maintain the microecology of the intestines and may have an impact on respiratory immunity and barrier function via the lung-gut axis, which ultimately contributes to IPF prevention [27]. In addition, yogurt is a plentiful source of diverse vitamins. Clinical evidence indicates that markers of disease severity and mortality in groups of IPF patients are closely connected to insufficient serum vitamin levels [28]. New mechanistic data demonstrates that Vit D obstructs the in vitro reaction of mouse lung fibroblasts to pro-fibrotic stimuli by disrupting signaling pathways regulated by TGFβ1-induced kinases [29]. Animal research findings suggest that VD3 improves survival in a bleomycin-induced model by reducing lung inflammation, hydroxyproline levels, collagen deposition, and apoptosis [30]. A study summarized potential mechanisms through which folic acid, a water-soluble B vitamin, plays a role in IPF pathophysiology, such as DNA repair, DNA methylation, and ROS pathways [31]. In conclusion, the influence of yogurt on IPF risk is complex, with its probiotics and nutrients suggesting preventive benefits, and further research is needed to fully understand these mechanisms.

Our study found that consuming dried fruit is a protective factor against IPF. Dried fruits have comparable nutritional content to fresh fruits, but the drying process leads to concentrated polyphenol content, which enhances antioxidant activity. Quercetin has the potential to rebalance disrupted redox equilibrium and alleviate inflammation by enhancing Nrf2 activity. Resveratrol, on the other hand, may mitigate bleomycin-induced pulmonary fibrosis by inhibiting HIF-1α and NF-κB expression [32, 33]. It is noteworthy that both the alkaline extract from the reticulated mandarin fruit peel and passion fruit peel extract had the potential to inhibit collagen synthesis, cross-linking, and deposition, thus showing promise as potential therapeutic agents for IPF [34, 35].

The study results showed that only the consumption of oily fish acted as a protective factor against IPF, while no direct link between non-oily fish consumption and the disease was found. This potential distinction may be due to the elevated content of omega-3 polyunsaturated fatty acids (PUFAs), specifically docosahexaenoic acid (DHA) and eicosapentaenoic acid (EPA), found in oily fish [36]. Studies have shown reduced levels of oleic acid surfactant phospholipids in IPF patients, indicating a potential preventive effect of increased PUFA consumption against IPF [37]. In an experimental study, it was found that the administration of DHA has the potential to mitigate paraquat-induced pulmonary fibrosis in rats by upregulating Smad 7 and SnoN protein levels [38]. Another study suggests that a diet high in EPA may effectively alleviate bleomycin-induced pulmonary fibrosis by modifying arachidonic acid-like metabolism [39]. This indicates that nanoemulsion fish oil supplements could potentially play a significant role in the future in preventing IPF through dietary consumption [40].

Compared with the consumption of oily fish, the consumption of beef was identified as a potential risk factor for IPF in this study. However, no definitive causal relationship was found for other types of red meat. This may be due to the high levels of saturated fat and cholesterol in beef, which, when consumed in excess, may lead to increased levels of TGF-β1 in airway epithelial cells. This cascade could further contribute to collagen deposition and an increase in the expression of pro-fibrotic factors, which are complex contributors to the progression of pulmonary fibrosis [41]. However, specific studies suggest that consumption of tallow (a lipid source) may result in increased pulmonary hydroxyproline levels and decreased lipid peroxidation following bleomycin administration, which could lead to a reduction in the severity of pulmonary fibrosis [42]. Therefore, it’s important to objectively evaluate the various roles that dietary factors may play in different contexts.

Regarding the association between alcohol consumption and the disease, the study found that both average weekly beer and cider consumption and alcoholic drinks per week were identified as risk factors for IPF. However, the failure to detect a causal relationship between red wine consumption and the disease may be due to the use of a single SNP as an instrumental variable. Chronic alcohol consumption has been associated with the development of IPF through several mechanisms. First, it increases the expression and activation of TGFβ1, which contributes to immune cell infiltration and collagen deposition in the lungs. These processes are thought to play a central role in triggering an abnormal fibrotic response, which is particularly evident in bleomycin-induced experimental acute lung injury [43, 44]. Second, alcohol consumption can lead to epithelial cell dysfunction, lung tissue remodeling, and oxidative stress, all of which may further contribute to the progression of alcohol-induced IPF [45–47].

Furthermore, it is essential to perform a detailed analysis of the strengths and limitations of this study. TSMR offers a significant benefit over traditional techniques such as self-reported questionnaires and dietary journals by establishing a more robust and dependable causal connection between diet and illness [48]. By using genetic variations as instrumental variables, MR effectively addresses issues related to confounding and reverse causation, improving the validity and accuracy of the findings. MR methods usually assume the independence of genetic variation effects on dietary factors from environmental influences, but gene-environment interactions can occur and impact the interpretation of the results [17]. As the number of GWAS meta-analyses increases, so does the risk of sample overlap between studies, which can result in biased outcomes [49]. Failure to correct for multiple hypothesis testing in this study reduces the ability to effectively assess false positive rates. Additionally, as the research focuses only on European populations and limited dietary categories, future studies should broaden the scope to include diverse diets and global populations.

## Conclusion

In summary, this study delved into the potential causal link between 29 common dietary habits and the risk of developing IPF. It revealed that the consumption of oily fish, yogurt, and dried fruits is associated with a reduced incidence of the disease, while the consumption of beef and alcoholic beverages poses a risk for IPF. In future studies, we will investigate a broader array of dietary choices and their potential mechanisms to establish evidence supporting scientifically informed dietary strategies for early IPF prevention.

### Supplementary Information

**Additional file 1: Supplementary Table 1.**  All Instrumental Variables used in this TSMR analysis.

**Additional file 2.** STROBE-MR Table.

## Data Availability

All data could be found in the IEU open GWAS project (https://gwas.mrcieu.ac.uk/).

## Abbreviations

IPF: Idiopathic pulmonary fibrosis
RCT: Randomized controlled trial
TSMR: Two-sample Mendelian randomization
FFQ: Food Frequency Questionnaires
IV: Instrumental variable
IVW: Inverse variance weighting
LOO: Leave-One-Out
PUFA: Polyunsaturated fatty acid
DHA: Docosahexaenoic acid
EPA: Eicosapentaenoic acid
